# Computational modeling of light processing in the habenula and dorsal raphe based on laser ablation of functionally-defined cells

**DOI:** 10.1186/s12868-024-00866-z

**Published:** 2024-04-16

**Authors:** Ruey-Kuang Cheng, N. Suhas Jagannathan, Ahmad Ismat Kathrada, Suresh Jesuthasan, Lisa Tucker-Kellogg

**Affiliations:** 1 Lee Kong Chian School of Medicine, Nanyang Technological University, 636921 Singapore, Singapore; 2 Centre for Computational Biology, and Duke-NUS Graduate Medical School Singapore, 8 College Road, 169857 Singapore, Singapore; 3 Program in Cancer and Stem Cell Biology, Duke-NUS Graduate Medical School Singapore, 8 College Road, 169857 Singapore, Singapore; 4 Department of Biomedical Engineering, National University of Singapore, 4 Engineering Drive 3, 117583 Singapore, Singapore; 5 Present Address: Neural Circuitry and Behavior Laboratory, Institute of Molecular and Cell Biology, A*STAR, 138673 Singapore, Singapore

**Keywords:** Neural circuits, Computational modelling, Neural network, Multilayer perceptron, Functional imaging, Two-photon microscopy, Light processing system, Pulsatile activation

## Abstract

**Background:**

The habenula is a major regulator of serotonergic neurons in the dorsal raphe, and thus of brain state. The functional connectivity between these regions is incompletely characterized. Here, we use the ability of changes in irradiance to trigger reproducible changes in activity in the habenula and dorsal raphe of zebrafish larvae, combined with two-photon laser ablation of specific neurons, to establish causal relationships.

**Results:**

Neurons in the habenula can show an excitatory response to the onset or offset of light, while neurons in the anterior dorsal raphe display an inhibitory response to light, as assessed by calcium imaging. The raphe response changed in a complex way following ablations in the dorsal habenula (dHb) and ventral habenula (vHb). After ablation of the ON cells in the vHb (V-ON), the raphe displayed no response to light. After ablation of the OFF cells in the vHb (V-OFF), the raphe displayed an excitatory response to darkness. After ablation of the ON cells in the dHb (D-ON), the raphe displayed an excitatory response to light. We sought to develop *in silico* models that could recapitulate the response of raphe neurons as a function of the ON and OFF cells of the habenula. Early attempts at mechanistic modeling using ordinary differential equation (ODE) failed to capture observed raphe responses accurately. However, a simple two-layer fully connected neural network (NN) model was successful at recapitulating the diversity of observed phenotypes with root-mean-squared error values ranging from 0.012 to 0.043. The NN model also estimated the raphe response to ablation of D-off cells, which can be verified via future experiments.

**Conclusion:**

Lesioning specific cells in different regions of habenula led to qualitatively different responses to light in the dorsal raphe. A simple neural network is capable of mimicking experimental observations. This work illustrates the ability of computational modeling to integrate complex observations into a simple compact formalism for generating testable hypotheses, and for guiding the design of biological experiments.

**Supplementary Information:**

The online version contains supplementary material available at 10.1186/s12868-024-00866-z.

## Background

Light enables vision and also influences non-visual systems such as mood and alertness [1, 2]. The impact of light on mood and alertness is important for human well-being, but the underlying neurobiology is only partially understood [3]. Zebrafish are simple vertebrates whose transparent tissues are amenable to optical imaging. Their complex behaviour (e.g., swimming, hiding, freezing) provide a way for studying mood, anxiety, alertness, and other phenomena. The habenula is an evolutionarily conserved region of the brain that is of central importance in mood disorders, and is involved in processing responses to light. The habenula plays a pivotal role in motor and cognitive behaviours, by influencing the release of important neuromodulators such as serotonin, dopamine, epinephrine and histamine [4–7]. In clinical studies, deep brain stimulation of the habenula has been successful at triggering remission of therapy-resistant depression [8]. The role of the habenula in light processing is not fully understood, but light-evoked activity has been detected in the habenula of several different species, including the rat [9], pigeon [10] and zebrafish [11, 12].

Although the zebrafish brain is far simpler than mammalian brains, the zebrafish habenula has strong homology to the mammalian habenula. Just as the mammalian habenula contains two subdomains (medial and lateral), the zebrafish habenula can be divided into two subdomains - the dorsal (dHb) and ventral (vHb) habenula. In rat [9] and pigeon [10], electrophysiological recordings demonstrated that light triggers both excitation and inhibition in the habenula. Evoked activity, either phasic or sustained, was detected in both medial and lateral subdomains. Similar results have also been shown in zebrafish [12].

The dorsal raphe (DR) nucleus, a major structure downstream of the habenula, also participates in regulating brain state in response to lighting conditions as shown by recent studies in which larval zebrafish prefer different lighting conditions after optogenetic manipulation of the raphe [13] or habenula [12]. Tracing experiments have suggested that the medial habenula can modulate DR activity via the interpeduncular nucleus [14, 15], while the lateral habenula has direct and indirect projections [16]. The roles of these different pathways in mediating the effects of light is unknown. One way to confirm causal relationships between anatomical regions of the brain is to perform localized perturbations, such as via two-photon laser ablation to lesion specific cells [17, 18]. Two-photon laser ablation can provide a causal relationship because it allows ablation of functionally specific cells, which cannot be easily done with a genetically encoded tool. Nevertheless, establishing neuronal connectivity is rarely simple, and the results of localized perturbations can be extremely non-intuitive to interpret directly.

Mathematical modeling is used in many scientific studies where experimental evidence provides non-obvious clues about underlying phenomena: the model provides an interpretation of the evidence (a candidate mechanism), while computation provides unbiased evaluation (across millions or billions of candidates) for whether the candidate interpretation is indeed consistent with the observations. Here, we investigated raphe response to light (i.e., calcium response of neurons in the dorsal raphe), immediately after functionally specific (ON or OFF) cells of the habenula were ablated. Specifically, we developed a two-layer feed forward neural network model, that used the habenula response to light as input to simulate the raphe response. The goal of this modeling is to provide a compact computational replica of the experimental findings, because summarizing and recapitulating a complex system can give insight into causal patterns and hypotheses for future experimental testing.

## Results

### The habenula displays a broad and dynamic response to irradiance change

**Fig. 1:**
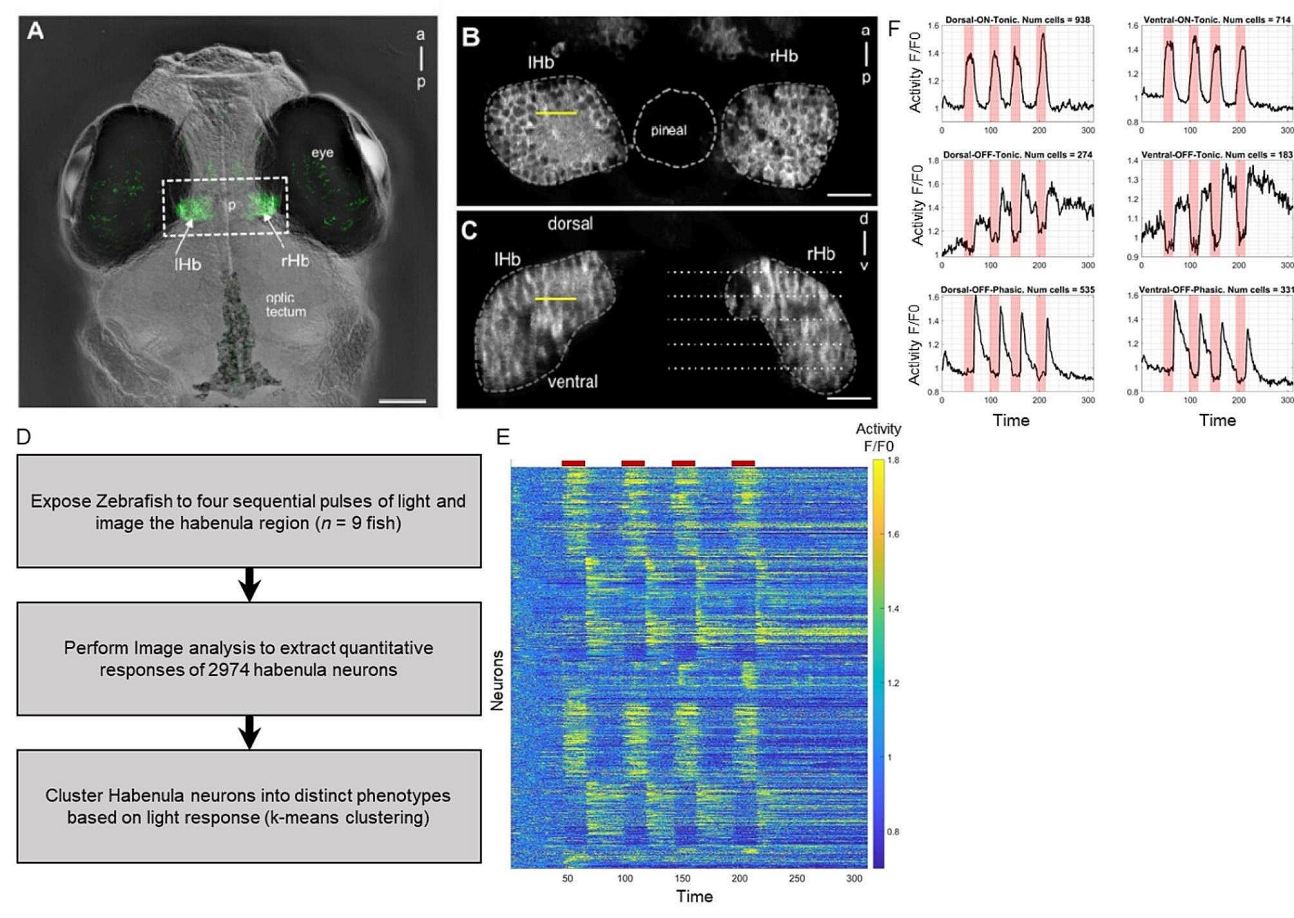
The habenula has multiple subtypes of cells that show differential response to light. (**A**) Dorsal view of the head of a live 7-day-old fish, with GCaMP3 expression in the habenula (arrows) under the control of the s1011t GAL4 driver. (**B**) A single two-photon slice through the dorsal habenula of the fish in panel A (boxed region). (**C**) A *yz*-reconstruction at the point indicated by the yellow line in panel B, showing a transverse view of the habenula. The dotted lines indicate imaging planes separated by 10 μm. The yellow line indicates the plane imaged in B. Dashed lines show the border of the habenula. (**D**) Workflow of habenula analysis (**E**) Heat map showing the activity of 2974 individual neurons (rows) across multiple light on and off cycles. Red bars on top correspond to the time period when light is switched on. As seen, some neurons show higher activity during light exposure (ON cells) and some neurons show higher activity after the light is switched off (OFF cells). (**F**) Response (y-axis) vs. time (x-axis) for habenula neurons that were clustered into subgroups depending on their response to light. Pink regions in each plot corresponds to the light exposure period. Seen here are the three subtypes in the dorsal habenula region (left) and three in the ventral habenula region (right). In total six subtypes were identified: D-ON-Tonic, D-OFF-Tonic, D-OFF-Phasic, V-ON-Tonic, V-OFF-Tonic, and V-OFF-Phasic. lHb: left habenula; rHb: right habenula; a: anterior; p: posterior. Scale bar = 25 μm

The zebrafish habenula consists of neurons surrounding neuropils that are innervated by afferent neurons [19–22]. We first characterized habenula activity evoked by pulses of light. Two-photon imaging was performed on a transgenic zebrafish line expressing the calcium indicator GCaMP3 throughout the habenula [23] (Fig. 1A). Resonant-scanning, combined with piezo-driven focusing, was used to record the activity of cells at multiple focal planes (Fig. 1B, C). With a step size of 10 μm, so that each cell would be sampled only once, most of habenula could be covered with 5 planes at a rate of 1 Hz. Habenula activity was monitored as the larva was exposed to 20- second pulses of blue light. We used relatively long pulses, rather than brief flashes, to allow longer characterization of responses. Steps involved in the analysis of habenula response to light are shown in Fig. 1D. In total, 2974 cells were identified in the habenula, and an analysis of their responses to light pulses showed the presence of multiple neuronal subtypes (Fig. 1E). We found neurons that showed excitatory response to light onset (the ON response) and also neurons that showed excitatory response to light offset (the OFF response). The finding here is consistent with previous findings using a short (1 s) red light pulse [11] and a 10-second blue light pulse [12].

We used the temporal light responses to group dorsal habenula (dHb) and ventral habenula (vHb) cells into individual clusters. The 2974 cells were first classified into dHb (1747 cells) or vHb (1227) region, depending on physical location. Then, k-means clustering was performed for each region (see Methods). Resulting clusters consisted of cells that were excited by light (ON cells), cells that were excited by subsequent darkness followed by sharply spiked response (phasic OFF cells), or cells excited by darkness followed by gradual decrease (tonic OFF cells). Both ON and OFF responses were seen in both dHB and vHb (Fig. 1F). This suggests that light OFF (or darkness) causes specific events in specific habenula cells, distinct from the ON cells. Thus, depending on their light response, the six habenula clusters identified were named D-ON-Tonic, D-OFF-Tonic, D-OFF-Phasic, V-ON-Tonic, V-OFF-Tonic, and V-OFF-Phasic. We refer to these six as the habenula subtypes.

### Raphe response is modulated differently by ablating different regions of the habenula

**Fig. 2:**
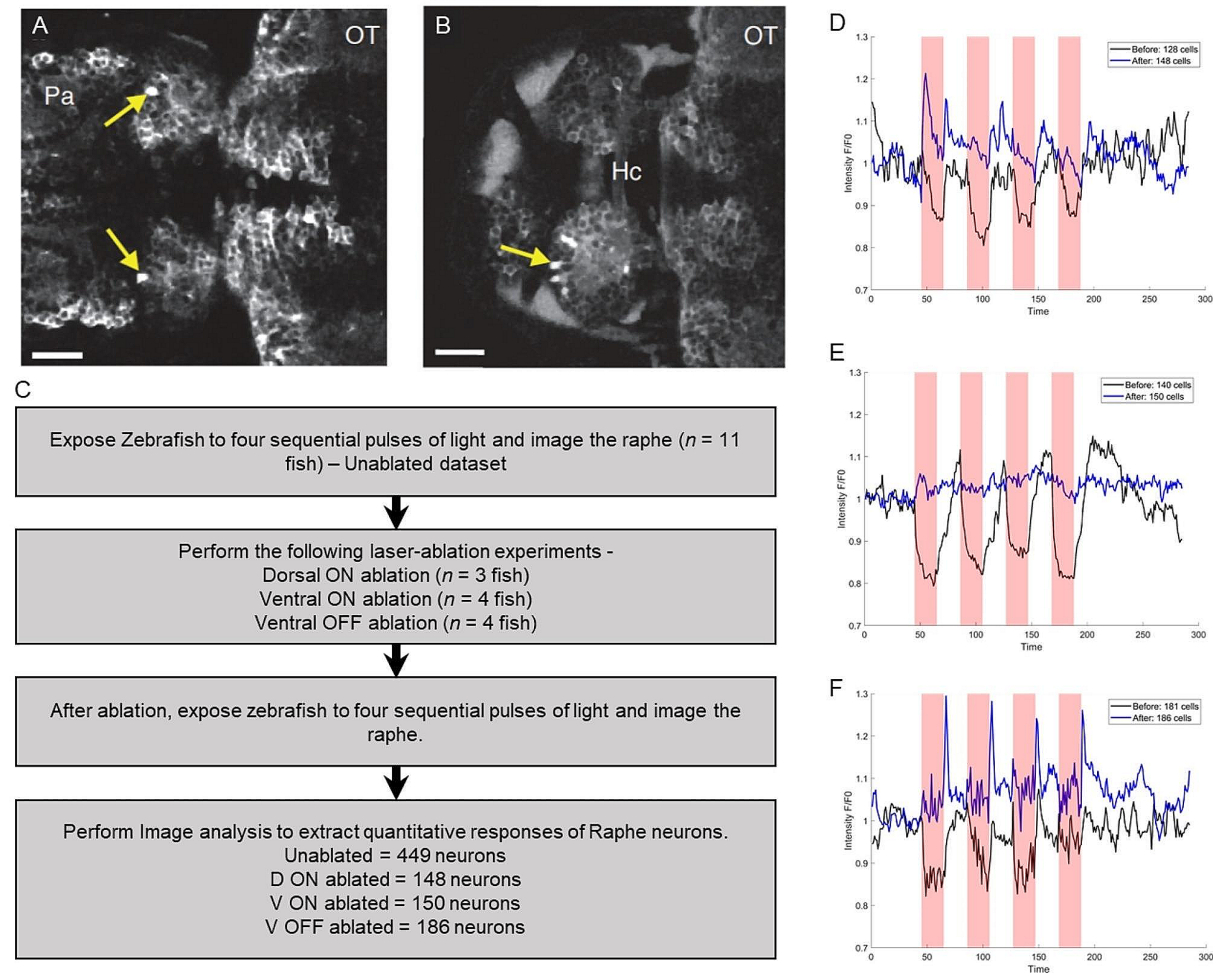
The effects of lesioning specific cells in the habenula on raphe response to irradiance change. (**A-B**) Examples of lesioning. The arrows indicate individual cells with elevated levels of intracellular calcium, following two-photon laser lesioning. (**C**) Workflow of raphe analysis. (**D-F**) The response of raphe (y-axis) vs. time (x-axis) when the habenula is intact (black curve) vs. when specific cells are lesioned in the habenula (blue curve). Red regions correspond to periods of light exposure. When the habenula is intact, the raphe is inhibited during light exposure in all three experiments. (**D**) The raphe shows inconsistent activation during light exposure when ON cells in the dorsal habenula (D-ON) are ablated. (**E**) The raphe is almost unresponsive when ON cells in the ventral habenula (V-ON) are ablated. (**F**) The raphe shows activation as soon as light is switched off, when the OFF cells in the ventral habenula (V-OFF) are ablated

Given the dynamic response in the habenula as shown in Fig. 1 and the persistence of dorsal raphe (DR) inhibitory response by light ON, as shown previously [13], we further tested if there is any functional link between the habenula light response and the DR inhibitory response by light ON. To test this, we used the technique of laser ablation or “lesioning” [12, 24], which allows localized disruption. In general, lesioning led to a sustained increase in GCaMP6f fluorescence throughout the targeted cell (Fig. 2A). Damage to fibers of passage was controlled for by imaging: namely, we only used lesions that did not lead to transient or sustained GCaMP6f fluorescence increase in regions away from the targeted site (e.g. in the contra-lateral habenula or pallium). There was no obvious change in the dHb response when cells in the ventral regions of the habenula were targeted, indicating that fibers of passage, which innervate the dorsal habenula, are not damaged. The workflow for our analysis of raphe response to light can be found in Fig. 2C.

In concordance to results from our earlier studies, we found again that with an intact habenula, the DR showed an inhibitory response during light exposure (black curves, Fig. 2D-F). When the laser was targeted to the left dorsal habenula neuropil regions that are activated by light ON, there was now light-evoked excitation rather than inhibition in the DR (Fig. 2D). Lesioning ON cells in the ventral region of the habenula led to a reduction in tonic activity in the DR, and few raphe cells showed any response to illumination change (Fig. 2E). When OFF cells were targeted in the ventral region, there was again a reduction in tonic activity of serotonergic neurons, accompanied by strong excitation upon transition to darkness (Fig. 2F). These lesion studies show that the distinct functional populations of the habenula, in response to light onset and offset, dynamically regulate response in DR serotonergic neurons. However, the nature of the habenula influence on the raphe was not clear from our experiments, and so we next developed a computational model.

### Model construction

**Fig. 3:**
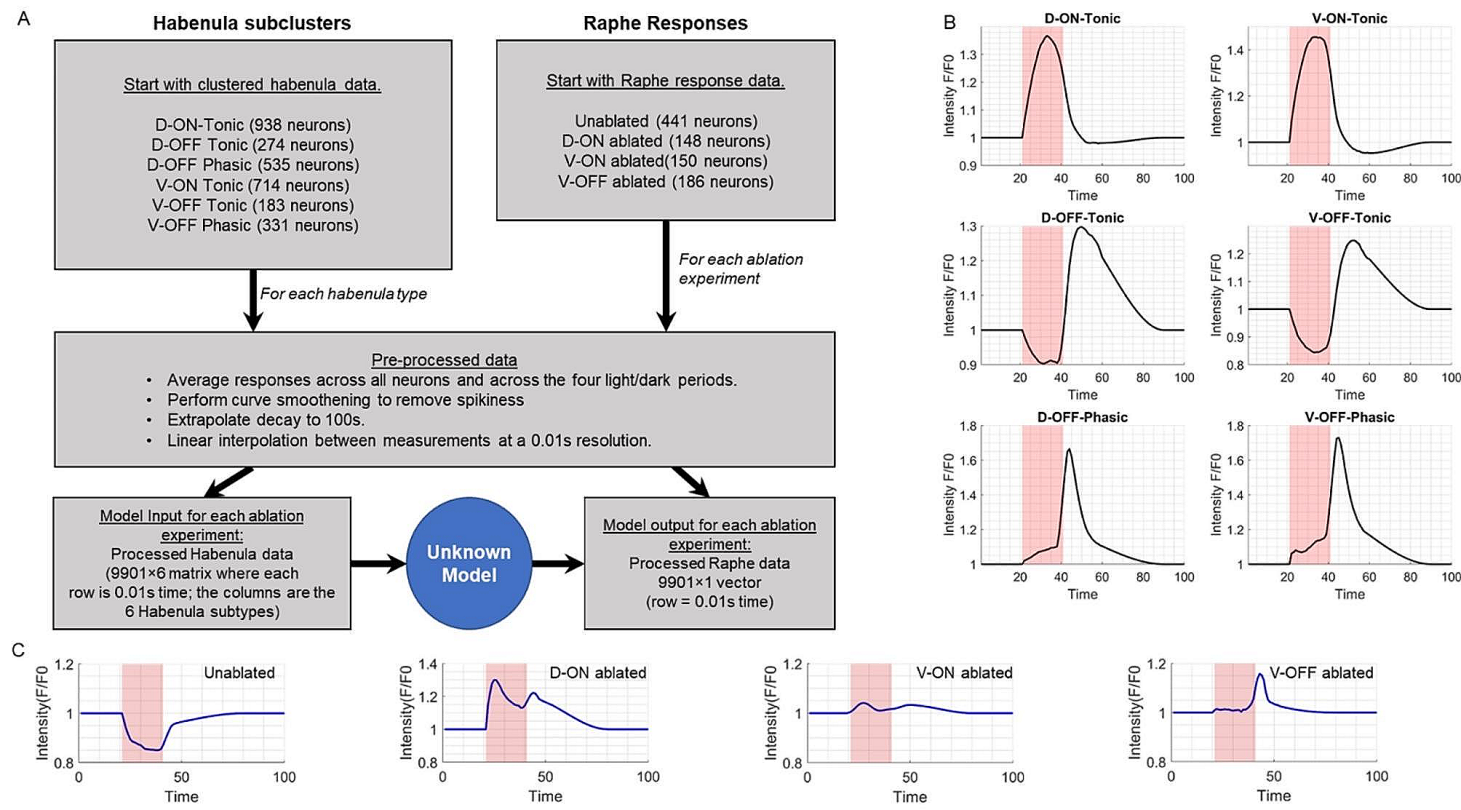
Characteristic responses of habenula and raphe to one light-dark cycle. (**A**) Schematic showing the workflow and data structures obtained from processing the raw neuronal activity data from the habenula and the Raphe. The “Unknown Model” represents an as-yet unknown model whose goal would be to use the habenula data as input to estimate raphe behaviour from the ablation experiments. (**B**) Characteristic responses of the six habenula subtypes to light exposure were obtained by averaging across the four light-dark periods. This was followed by padding and interpolation to extend the response trajectory to 100s so all neurons would reach the baseline, followed by smoothing. (**C**) Characteristic responses of raphe neurons to unablated conditions or ablation experiments were obtained from the response of individual raphe cells to the first light exposure and following dark period

The goal of the modeling is to recapitulate the behavior of raphe (the output) in response to the habenula (the input) when different parts of the habenula are ablated or not. We curated the input to the modeling as follows. For each of the six habenula subtypes (Fig. 1F), we first created characteristic responses over a 100 s time frame that included a single 20s light exposure window (see Methods and Fig. 3A). After smoothing these curves, we used linear interpolation to obtain values at a resolution of 0.01s (the experimental measurements were taken at 1 s intervals). This resulted in a 9901-long vector for the neuronal response for each of the six habenula subtypes, for each of the four experimental conditions (3 ablations and 1 non-ablation condition). When a particular habenula subtype was ablated, its neuronal response was set to zero for that experimental condition. The 9901-long vectors of neuronal response were gathered for all six habenula subtypes and all four ablation or non-ablation conditions into a 39,604⨯6 matrix. Each column corresponds to one habenula subtype, and each row corresponds to the habenula response from time *t* = 1s to *t* = 100s in increments of 0.01s, concatenated across four experimental conditions. This habenula matrix is the input for our efforts to model how the raphe responds to the habenula (Fig. 3B).

For the raphe region of the brain, the same processing steps (averaging, smoothing, interpolation) were performed to obtain a raphe response profile under each of the four experimental conditions: unablated, D-ON ablated, V-ON ablated and V-OFF ablated. This resulted in a vector of size 39,604⨯1 for the raphe output dataset (the target output for our modeling, Fig. 3C).

We first tried to obtain mechanistic models of habenula-raphe interactions by developing multiple Ordinary Differential Equation (ODE) models that employed incoherent feedforward loops. The ODE models used biologically-informed networks of neuronal activation and inhibition, similar to those in Additional File 2. However, our ODE modelling efforts failed to recapitulate raphe responses from habenula inputs.

**Fig. 4:**
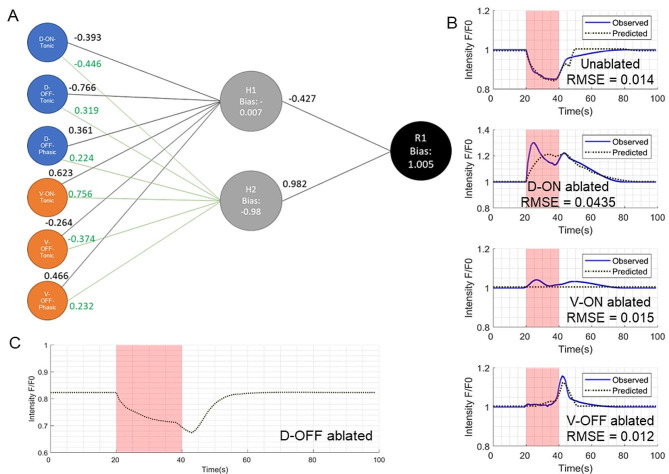
Neural network model and estimated raphe behavior. (**A**) The final neural network model that was able to recapitulate raphe behaviour from habenula input. The first layer of the network has six nodes corresponding to the six habenula subtypes. The middle layer has two nodes and the final output layer has one node whose output is the estimated raphe behaviour. The numbers on the edges represent the weights of the edge. (**B**) Observed raphe behaviour (blue) and model-estimated raphe behaviour (dotted gray) for the unablated and the ablation experiments shows good fit (RMSE of the fit shown on the plots) (**C**) Model-estimated raphe response for a potential D-OFF ablation experiment

We then proceeded to implement simple multi-layer perceptron (MLP) feed forward neural networks (NN). Such networks would have an input layer with six nodes (the six habenula subtypes) and an output layer with one node (raphe response). Between these layers would be an intermediate hidden layer that would assimilate the habenula inputs and pass a signal to the raphe. In the simplest form, this hidden layer would have a single node and this design of the neural network also failed to recapitulate raphe responses. As the next simplest design, we tried a model with two nodes in the hidden layer, and this simple model was able to fit the observed raphe responses to a satisfactory degree, as estimated by the root mean square error (RMSE) between the experimentally observed and model-estimated Raphe behaviors. The weights and biases of the best fit model can be seen as arrow labels on Fig. 4A. The fits of model-estimated vs. observed raphe responses and the RMSE values are shown in Fig. 4B. The RMSE values for the unablated case, the V-ON ablated case and the V-OFF ablated case were all low and comparable (0.014, 0.015 and 0.012 respectively). The RMSE for the D-ON ablated experiment was comparatively higher at 0.0435, but still included a neuronal activity peak during the light ON period and a second peak once light was switched OFF, as observed in the experiments. The overall RMSE for all four ablation experiments was found to be 0.0246.

Because this is a simple network, the edge weights can also be used to draw inferences about neuronal influences over other neurons in the network, hinting towards the beginnings of mechanistic understanding. The weights suggest that the D-ON-Tonic cells in the habenula have an inhibitory effect on both intermediate nodes, while the V-ON-Tonic cells have an activating effect. It is important to note that since the corresponding neurons in dorsal and ventral regions (ON-Tonic, OFF-Tonic and OFF-Phasic) have mostly similar (but not identical) behaviors to light (Fig. 2), it is possible to get similar (but not identical) raphe behavior in the unablated experiments by assigning the weights of the Dorsal nodes to the ventral and vice versa. However, this would not fit the observations from the D-ON and V-ON ablation experiments. It is interesting that the two ON-Tonic nodes have opposing effects on the intermediate nodes. In contrast both D-OFF-Phasic and V-OFF-Phasic nodes have only activating effects on both intermediate nodes. Also, while the V-OFF-Tonic node has an inhibitory effect on both intermediate nodes, the D-OFF-Tonic node has an activating effect on one intermediate node and an inhibitory effect on the other intermediate node.

Finally, our model was also used to estimate the potential raphe response to a hypothetical D-OFF ablation experiment (Fig. 4C). Such an experiment in the future may be used to validate/ fine tune the parameters of the model. To test the robustness of our model we also performed ten-fold cross validation on our dataset. Briefly, for each ablation experiment, we split the raphe datasets into ten non-overlapping sets of neurons. Each set was taken to be the validation set for one iteration of the cross validation, while the other nine were taken as the training set. The average raphe responses were computed for the training and validation datasets. An MLP was fit to the training set and the resulting model (the “best-fit MLP”) was evaluated using the RMSE relative to the training set and relative to the validation set. This was repeated for ten iterations. The range of training and test errors over the ten-fold cross validation can be found in Additional File 3.

### Interpreting the NN model parameters

**Fig. 5:**
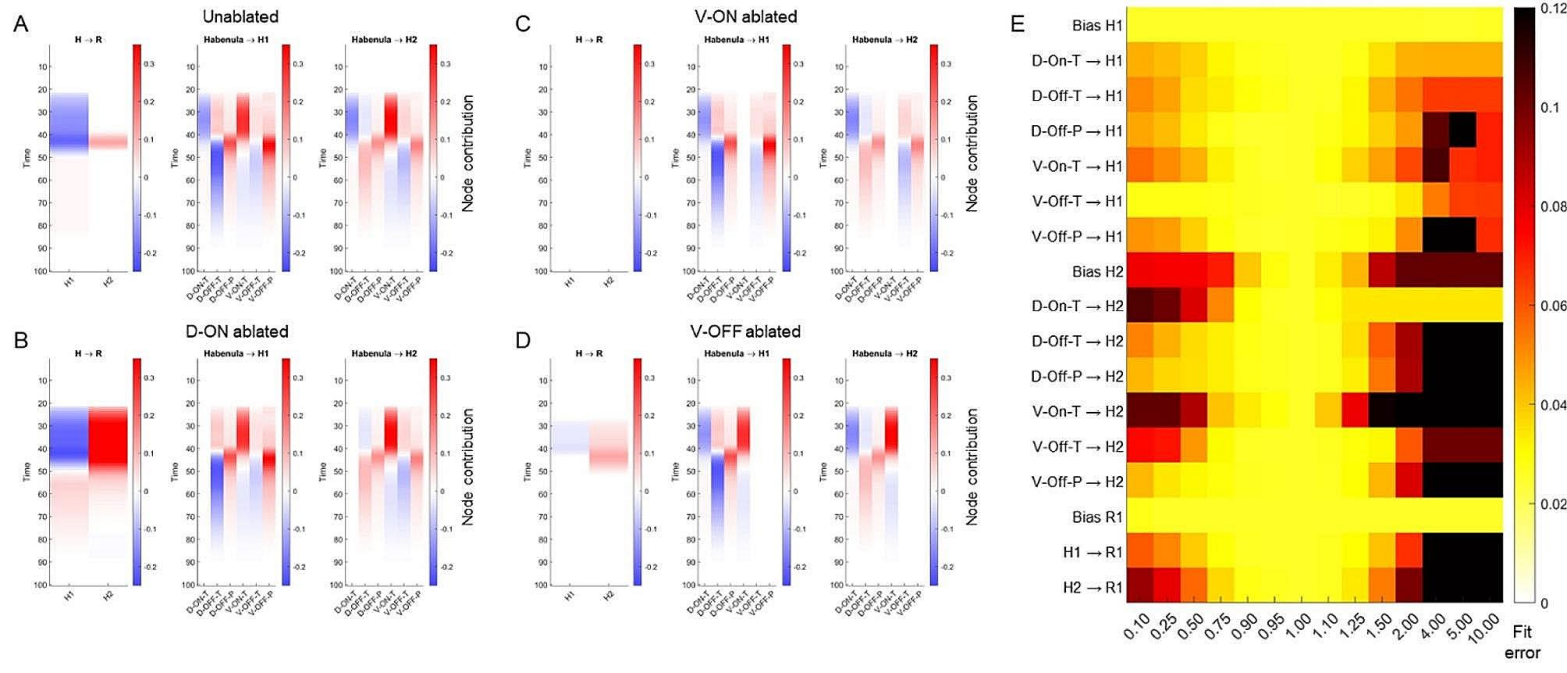
Contributions of each neural network node to the Raphe response. (**A-D**) Contributions (y-axis) of each NN node (x-axis) to the nodes in the successive layer over 100s (y-axis). In each panel, the leftmost column shows the contribution of H1 and H2 nodes to the Raphe output (after scaling by the respective Layer 2 weights). The middle panel shows the contribution of the six Habenula subtypes to the H1 node (after scaling by the respective Layer 1 weights), and the right most column shows the contribution of the same six habenula subtypes to the H2 node (after scaling by the respective Layer 1 weights). The contribution intensities are indicated by the color scheme ranging from negative/inhibition (blue) to positive/activation (red). (**A**) shows the node contributions in the unablated experiment, (**B**) in the D-ON ablation experiment, (**C**) in the V-ON ablated experiment and (**D**) in the V-OFF ablation experiment. In the ablation experiments, the contributions of the ablated neurons can be seen to be zero. (**E**) shows the sensitivity of the final estimated Raphe output to changes in each NN parameter. The intensities show the fit error computed as the RMSD between the model-estimated and observed Raphe responses across the unablated and three ablation experiments

To seek mechanistic understanding from our best fit neural network (NN) model, we computed the contribution of each node in our model, to nodes in the successive layer (Fig. 5A-D). According to our model, in the unablated case, the inhibitory phenotype shown in the raphe response to light activation is mostly driven by an inhibitory effect of the H1 node on the output raphe node. This can be seen by the negative values of H1→R in the leftmost column of Fig. 5A between 20 and 40s (period when light is on). It is also important to note that the weight of the edge between H1 and R in our model is negative, and so strong activators of the H1 node will cause strong inhibition of the raphe node. Looking at the contribution of individual habenula subtype nodes to H1, we see that H1 activation during light is mostly due to strong activation by the V-ON-Tonic cells (middle column, Fig. 5A). In contrast, the recovery of this inhibitory phenotype after light is switched off, is due to activation of the raphe node by the H2 node. This can be seen from positive values of H2→R between time 40–50 s in the leftmost column of Fig. 5A.

In the D-ON ablated case, we see that raphe activation during light activation occurs as a competition between inhibiting H1 and activating H2 nodes, resulting in overall activation of the raphe node (leftmost column Fig. 5B). As earlier, this is a consequence of V-ON-T activation of the H1 and H2 nodes. In addition, the ablation of D-ON results in the removal of D-ON inhibition of H2, which also contributes to activation of the R node by H2 (as opposed to no effect in the unablated case). In the V-ON ablated case, the lack of strong activation of H1 and H2 nodes (seen earlier) is now absent, and hence is responsible for an overall loss of signal from H1 and H2 to the R node (Fig. 5C). Finally in the V-OFF ablated case, the strong phasic response after light offset is driven mostly by H2 activation of the R node, as evidenced by the positive values of H2→R between time 40–50 s in the leftmost column of Fig. 5D. In addition, ablation of the V-OFF-Phasic node results in removal of the inhibitory effect due to V-OFF phasic activation of H1 node in the unablated case.

### Model sensitivity analysis

To obtain the sensitivity of model estimation to changes in each parameter of the NN model, we individually scaled each NN parameter by a factor between 0.1 and 10 (0.1x– 10x) and compared the model simulations against experimental observations of raphe behavior. For each ablation experiment, the error is compared as root mean square deviation after the simulated raphe response has been translated to have a median response of 1 over the first 10s (out of the 100s window). As seen in Fig. 4C, the NN model was more sensitive to changes in some parameters and less sensitive to others. For example, the model is relatively less sensitive to changes in the weight of the edges from D-ON-Tonic to the H1 and H2 nodes. In contrast, the model is much more sensitive to changes in the edges from V-ON-Tonic to H2, which is unsurprising, considering the magnitude of the contributions of V-ON-Tonic to raphe responses, seen in Fig. 5A-D. Also, as a general trend, higher error values are found when parameters are scaled up rather than scaled down. Furthermore, the model is less sensitive to changes in edges going into the H1 node than into the H2 node.

## Discussion

Typically, ablation of upstream neuronal regions causes downstream regions to lose responsiveness to external stimuli. Remarkably, in our experiments, lesioning different types of functionally-specific neurons in the habenula caused the downstream neuronal responses in the dorsal raphe to change in several unexpected ways– from inhibitory to excitatory, from light-excitatory to dark-excitatory, and from tonic to phasic. Our work used brief exposures to light and dark in larval zebrafish, because light and dark are strong stimuli for these brain regions and therefore aid dissection of neural connectivity. Another reason we used light and dark is because they are evolutionarily connected to predators and danger response. In humans, danger response is linked to psychiatric conditions such as anxiety and depression, and the human habenula is known to have an influential role in anxiety and depression.

Previous work showed that neural activity in the dorsal raphe is generally inhibited by light [13], and we also found that dorsal raphe neural activity (measured by calcium fluorescence intensity change) was negatively correlated with light exposure. We then identified different populations of habenula cells (ON and OFF cells) that were positively or negatively correlated with light. We used laser ablation to disable specific types of neurons in the habenula, specifically the ON-cells or specifically the OFF- cells. The raphe response to light and dark, after each type of ablation, was altered in remarkable ways which contain intricate information about how the dorsal raphe is affected by different types of neurons in the habenula.

To characterize and analyze this unexpected diversity of responses, we analyzed neuronal imaging and constructed a simple multilayer perceptron model that was able to recapitulate raphe responses from habenula input. We chose an MLP model for our estimations because compared to other statistical algorithms for prediction, a simple MLP model allowed both high regression accuracy and the ability to draw insights from the model parameters. Our computational simulations using the MLP model mimic the biological observations by producing four different states of the DR: (1) inhibited by light; (2) spontaneously active regardless of irradiance; (3) excited by light and (4) excited by darkness. While we do not claim that the model in Fig. 4A is the only model that is capable of recapitulating raphe responses from habenula inputs, it could be one among a set of similar equally-likely models. Such a set of equally-likely models would include uncertainty about the parameter values as well as mechanistic uncertainty. These variations cannot be disambiguated with the available data, but we can confidently assert that a small number of connections (as in Fig. 4A) is *sufficient* to explain the flipped responses seen in the ablation experiments seen in Fig. 2. The problem of choosing among possible models depends on whether the goal is fitting or prediction. Our primary goal is to describe the data rather than to generalize to different cases, but our model has parameters that might predict raphe behavior in unknown cases, so it illustrates one plausible scenario with a logical explanation for interpretation of the experimental results. When additional experiments are performed in the future, the resulting data can further improve the modeling. While we did perform ten-fold cross validation with our model, there were challenges with such validation. Since the input and output data are representations of average behavior of habenula and raphe, there is no strict one-to-one correspondence between the input and output instances. For true one-to-one correspondence, the habenula and the raphe responses would need to be measured in the same fish, which is not feasible with our ablation experiments. Furthermore, since each individual row or “instance” in the input and output corresponds to habenula/raphe behavior at time intervals separated by just 0.01s, it is very likely that the input and output values of any randomly chosen validation set would have very close neighbors (in value) in the training set. This defeats the purpose of a random validation set, as these values are not independent of each other. Hence, we chose to perform 10⨯ cross-validation by computing average behavior from a subset of raphe neurons, while using the average of the left-out neurons as the validation (Additional File 3).

Upon deeper inspection of the weights and the contributions of each node in our neural network to the final raphe response, we observed that most of the interesting phenotypes seen with the raphe response to light are dominated by the activity of V-ON-Tonic nodes and its activation of the H1 and H2 nodes, with smaller contributions from the other habenula subtypes. If our modelling estimations are accurate, that would also imply the presence of biological equivalents of the H1 and H2 nodes, which act as intermediates between the habenula and raphe. While previous studies have suggested the rostromedial tegmental nucleus as one such mediator [16], future experiments can help identify the anatomical region and the precise set of neurons involved in this mediation.

In summary, this study used computational modeling to delineate specific hypotheses about the interplay between the dorsal and ventral habenula during raphe response to light and dark, with systems-level correlations emerging from analysis of the best fit model. Computational modeling is particularly valuable in the field of neuroscience because even a single lesion in the brain can yield rich and non-intuitive phenotypes. Our model reveals that a simple network can create a variety of complex behaviors, and can explain the seemingly paradoxical observations observed with ablation experiments. Other aspects of the model, such as the hypothesized nature of each functional connection and the relative strength of each effect are more speculative, and their usefulness is more likely to lie in the holistic illustration of a plausible scenario rather than a literal specification of calcium regulation in the neurons. Qualitative modeling can shed light on confusing phenomena and guide the choice of validation experiments, while providing testable hypotheses for future experiments.

## Conclusion

Because the habenula has a causal influence on anxiety/depression disorders, there is urgent need to map functional connectivity from the habenula to the raphe at the level of neurons or neuron clusters. When neurons in specific regions of the habenula were ablated, neurons in the dorsal raphe showed reproducible patterns of change in how they responded to light and dark. Response patterns exhibited a surprising variety of qualitative changes, depending on which habenula site was ablated. Computational modeling was undertaken because simulating this variety could aid the design of future experiments and the disambiguation of potential mechanisms. An artificial neural network was trained by statistical machine learning and was successful at recapitulating the observed range of response profiles. In other words, the surprising variety we observed could be captured using a simple, compact formalism. Future work should continue to upgrade models of the habenula until both the model mimics the microanatomy in not just its outputs but also its internal formalisms. Inevitable future growth in data availability and computational resources will expand modelling research toward comprehensive integration of neuroscience experimentation and simulation. Such integration will be necessary for the overarching biomedical objective, which is mapping the functional connectivity of zebrafish neuronal structures that are homologous to structures implicated in human psychiatric disorders.

## Methods

### Experimental methods

#### Fish lines

Experiments were performed in accordance with guidelines issued by the Institutional Animal Care and Use Committee (IACUC) of the Biological Resource Centre at Biopolis, Singapore. Zebrafish (*Danio rerio*) lines used for this study were: *Tg(tph2:GAL4, UAS: Kaede)*^*y228*^ [25], *GAL4s1011t* [26] *and Tg(elavl3:GCaMP6f)*^*a12200*^.

#### Two-photon calcium imaging

Zebrafish larvae (aged 5–10 days-post-fertilization, dpf) were immobilized in mivacurium (1.5 mg/ml) and embedded in low-melting temperature agarose (2.0% in E3) in a glass-bottom dish (Mat Tek). They were imaged on a Nikon two-photon microscope (A1RMP), using a 25x water immersion objective (NA = 1.1). The femtosecond laser (Coherent Vision II) was tuned to 920 nm for GCaMP6f imaging. Stacks were collected in resonant-scanning mode with 2x pixel averaging. The sample size was based on [11]. Blue light stimulus was generated by 5 mm blue LEDs (458 nm peak emission), which was powered by a 5 V TTL signal from a control computer and synchronized with image capture using a National Instruments DAQ board, controlled by the Nikon Elements software. Each light pulse was 20 s long and followed by 20 s dark with a total of 4 pulses of light. Light intensity at the sample was 0.13 mW/cm^2^.

#### Laser ablation

Larval zebrafish (6–8 days post fertilization, dpf) were subjected to laser ablation for ON or OFF cells in the habenula, as described in [27]. Briefly, *Tg(Elavl3:GCaMP6f)* larvae were anaesthetized and then mounted in 2% low melting temperature agarose. After identifying habenula ON and OFF cells, lesions were conducted via several pulses (100–500 msec) of the femto-second laser (960 nm). Lesioning was monitored by time-lapse imaging before and after each pulse, and was terminated when there was a localized increase in GCaMP6f fluorescence. Animals were discarded if lesioning caused bursting of blood vessels in the habenula.

### Image analysis

#### Initial data pre-processing

All image processing steps were performed following the same protocol as our earlier work [27]. In short, raw images obtained were first registered to correct for any vertical/horizontal movement artefacts using cross-correlation. Then, a median filter of size 3 was applied to remove noise. A darker region outside the region of interest was chosen as the background and subtracted from the image to remove background noise. Non-linear trends in the data were detrended using polynomials of order 2–5. Data was then normalized into Z-scores by subtracting the overall mean and dividing by the standard deviation. A rolling window average was then used to smooth noisy traces where necessary. Where possible, cells were segmented (see below) or images were directly analysed as pixels (using the Thunder platform [28] for fast pixel-based clustering and factorization).

#### Cell segmentation

Each stack was scaled 2x in imageJ (RRID: SCR_003070), then maximally projected to a single image, which was then subjected to a minimum filter and unsharp mask to sharpen the boundary of cells. ROIs were identified using the “find maxima…” command, as a way to localize regional darkest point as the center of each ROI. The boundary of the ROI was outlined by “analyze particle…” that connects bright pixels into mosaic-like tessellated plane, encircling each darkest point. Each ROI was then numbered sequentially using the ImageJ ROI Manager and mapped back to the original despeckled image stack. Manual segmentation was done here to delete extraneous ROIs outside the habenula or dorsal raphe and to encircle cells that were not detected by the algorithm (< 10% of total ROIs). In the last step, “Set measurements…” and “measure” in ImageJ provided the mean fluorescence value of all pixels within each ROI across the entire image stack and the x-y coordinates of each ROI. Time-lapse series in which z drifting occurred were excluded, as in this case ROIs could not be defined.

### Statistical and modeling methods

#### Classification of the habenula cells

The habenula was segmented and then, to identify evoked responses to light, the spatiotemporal calcium dataset was subjected to k-means clustering as described in [27]. Habenula cells were classified into being dorsal or ventral depending on their location (position in the Z-stack), and k-means clustering was performed individually for cells in each region, as in our earlier studies [13, 27], to find cells that responded similarly to the light pulses. The clustering method was k-means and the distance measure was correlation, and this configuration ran quickly and was able to recapitulate well-established classifications of neuronal phenotype such as tonic and phasic. Not knowing *a priori* the ideal number of neuronal clusters, we ran k-means clustering with different k values from 2 to 10 and compared results. For each region (dorsal/ventral), we manually inspected the clustering results and judged the optimal k to be three (see Additional File 1 for clustering results for k values 2–5).

#### Defining model input and output

For each of the six habenula subtypes identified via k-means clustering, we first obtained a mean response for a 40 s window that comprised of a 20 s light period followed by a 20s dark period. This response was obtained by averaging across four subsequent light/dark cycles for each cell, and then computing the mean response of all cells in each cluster for this window. Although at the end of the 40 s window a new light cycle was initiated in the experiments, we observed that some phenotypes had not yet decayed to the baseline. Hence to simulate complete response for each habenula subtype, we first padded the response on each side as follows. For the time before light exposure, we appended a 20s period of baseline activity, simulating unexcited neurons. For padding after the 40s window, we used PCHIP (Piecewise Cubic Hermite Interpolating Polynomial) interpolation to extend the response for 40 more seconds, bringing the total to 100s for each habenula cluster (20s unexcited + 20 s light exposure + 60 s decay to baseline). These 100s habenula responses were then smoothed to remove noise and were used as input to our model.

Similarly for the dorsal raphe, we constructed a mean 100s behavior for raphe response to each ablation experiment as follows. Since the raphe response across subsequent light/dark cycles were not consistent for each cell unlike the habenula, we used only the response to the first light exposure to obtain mean 40s response for each experiment. These were then padded and smoothed similar to the habenula data to obtain 100s responses for the raphe. This was used as the “true” output against which our model to fit its simulated responses.

#### Neural network model

To simulate raphe responses from habenula input, we used a simple multilayer perceptron (MLP) neural network (NN). The network had 6 input nodes (corresponding to each habenula subtype), a hidden layer with two nodes and a third layer with one output node. All nodes in the hidden and output layer used a Rectified Linear Unit (ReLU) activation function. Any other hyperparameters were either set to default values (e.g., learning rate), or were not included (e.g., dropout). Earlier attempts with ordinary differential equation models employing incoherent feedforward loops, or an MLP with a one-node hidden layer proved insufficient to recapitulate raphe behavior from habenula input. The final NN model was implemented and fitted using the Keras (v2.2.4-tf) and TensorFlow (v2.1.0) APIs in Python 3.7.6.

### Electronic supplementary material

Below is the link to the electronic supplementary material.

Supplementary Material 1

Supplementary Material 2

Supplementary Material 3

## Data Availability

The images obtained from Zebrafish experiments can be found at the Figshare database (DOI: 10.6084/m9.figshare.21721058). The codes used in analysis can be found on github at the URL https://github.com/nsuhasj/habenulaRaphe/. Any other datasets used and/or analysed during the current study are available from the corresponding author on reasonable request.

## Abbreviations

DAQ: Data Acquisition
dHb: dorsal habenula
D–OFF: Dorsal OFF cells
D–ON: Dorsal ON cells
DR: Dorsal Raphe
LED: Light emitting diode
MLP: Multi–layer perceptron
NA: Numerical aperture
NN: Neural Network
ODE: Ordinary Differential Equation
PCHIP: Piecewise Cubic Hermite Interpolating Polynomial
ReLU: Rectified Linear Unit
RMSE: Root mean square error
ROI: Region of Interest
TTL: Transistor–Transistor Logic
vHb: ventral habenula
V: OFF–Ventral OFF cells
V: ON–Ventral ON cells

## Declarations

### About this supplement

This article has been published as part of BMC Neuroscience Volume 25 Supplement 1, 2024: The 21st International Conference on Bioinformatics (InCoB2022): neuroscience. The full contents of the supplement are available online at https://bmcneurosci.biomedcentral.com/articles/supplements/volume-25-supplement-1.
