# Supplementary material for: Computational modeling of light processing in the habenula and dorsal raphe based on laser ablation of functionally-defined cells
Source: BMC Neurosci. 2024 Apr 16;25(Suppl 1):22. doi: 10.1186/s12868-024-00866-z (PMC11022313; doi:10.1186/s12868-024-00866-z)
Supplement: Supplementary file 1 — Supplementary Material 1 [file 12868_2024_866_MOESM1_ESM.pptx]

## Slide 1
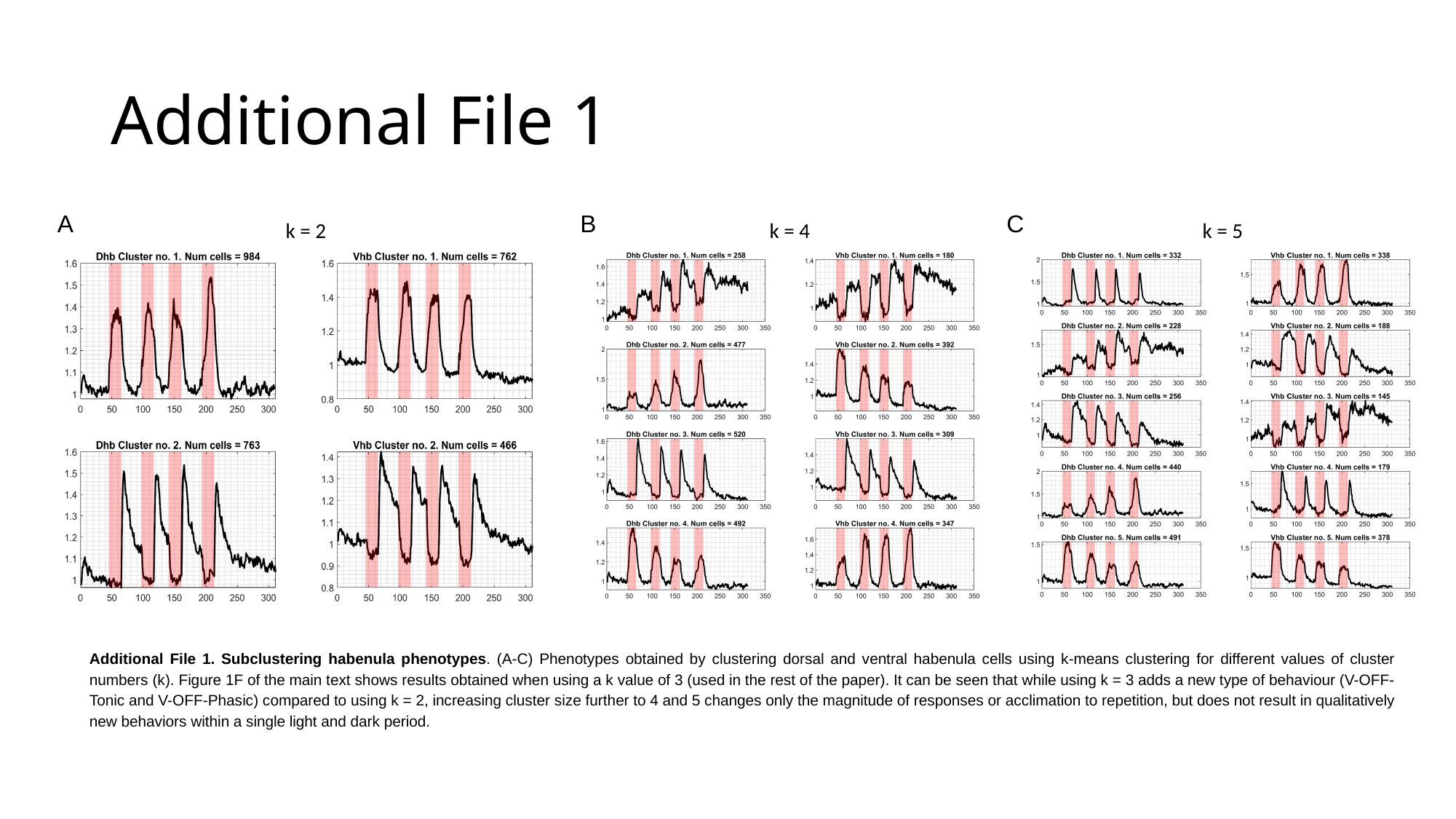

# Additional File 1
A
B
C
k = 2
k = 4
k = 5
Additional File 1. Subclustering habenula phenotypes. (A-C) Phenotypes obtained by clustering dorsal and ventral habenula cells using k-means clustering for different values of cluster numbers (k). Figure 1F of the main text shows results obtained when using a k value of 3 (used in the rest of the paper). It can be seen that while using k = 3 adds a new type of behaviour (V-OFF-Tonic and V-OFF-Phasic) compared to using k = 2, increasing cluster size further to 4 and 5 changes only the magnitude of responses or acclimation to repetition, but does not result in qualitatively new behaviors within a single light and dark period.
