# Supplementary material for: Computational modeling of light processing in the habenula and dorsal raphe based on laser ablation of functionally-defined cells
Source: BMC Neurosci. 2024 Apr 16;25(Suppl 1):22. doi: 10.1186/s12868-024-00866-z (PMC11022313; doi:10.1186/s12868-024-00866-z)
Supplement: Supplementary file 2 — Supplementary Material 2 [file 12868_2024_866_MOESM2_ESM.pptx]

## Slide 1
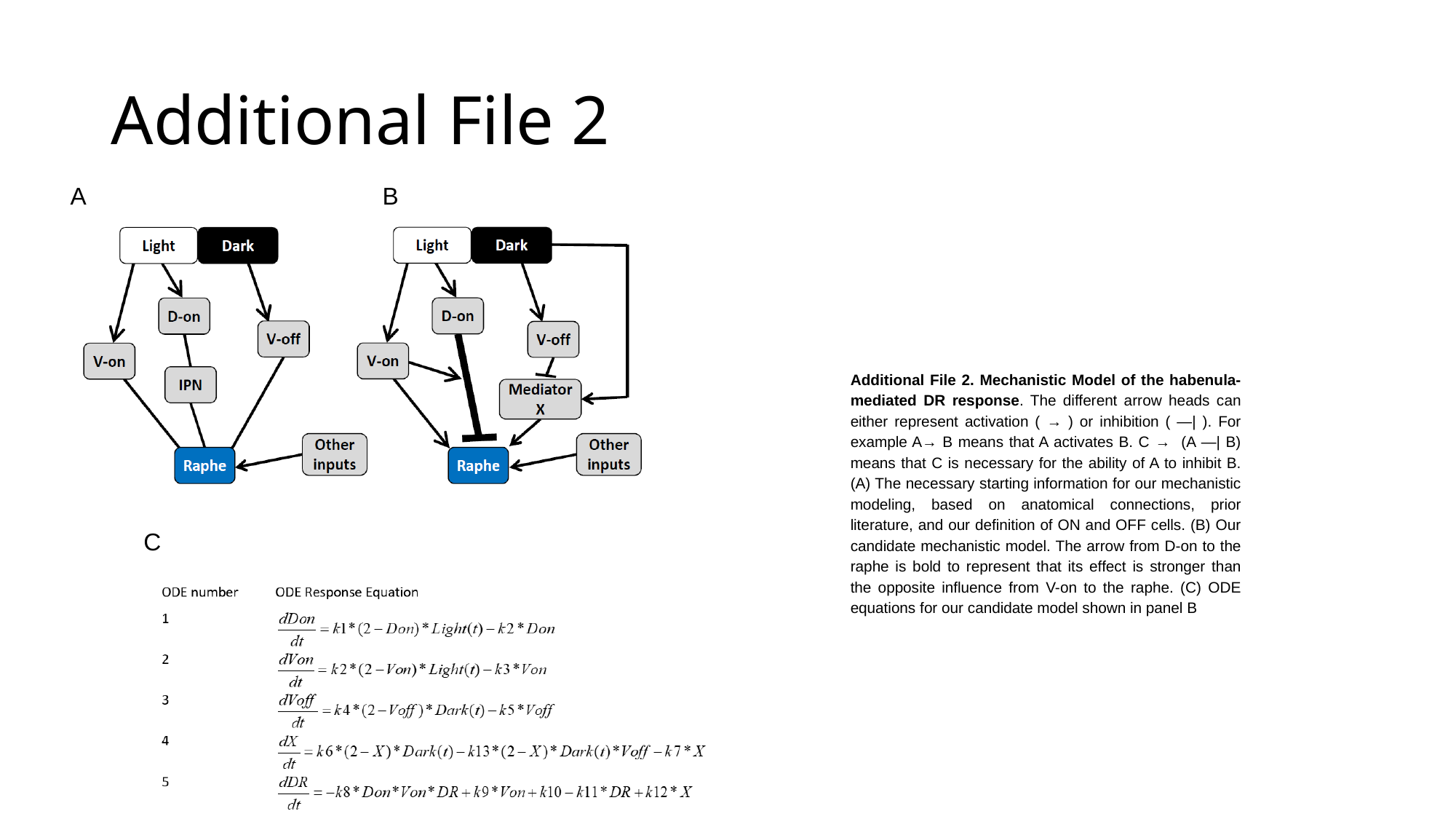

# Additional File 2
A
B
C
Additional File 2. Mechanistic Model of the habenula-mediated DR response. The different arrow heads can either represent activation ( → ) or inhibition ( ―| ). For example A→ B means that A activates B. C → (A ―| B) means that C is necessary for the ability of A to inhibit B. (A) The necessary starting information for our mechanistic modeling, based on anatomical connections, prior literature, and our definition of ON and OFF cells. (B) Our candidate mechanistic model. The arrow from D-on to the raphe is bold to represent that its effect is stronger than the opposite influence from V-on to the raphe. (C) ODE equations for our candidate model shown in panel B
