# Supplementary material for: Computational modeling of light processing in the habenula and dorsal raphe based on laser ablation of functionally-defined cells
Source: BMC Neurosci. 2024 Apr 16;25(Suppl 1):22. doi: 10.1186/s12868-024-00866-z (PMC11022313; doi:10.1186/s12868-024-00866-z)
Supplement: Supplementary file 3 — Supplementary Material 3 [file 12868_2024_866_MOESM3_ESM.pptx]

## Slide 1
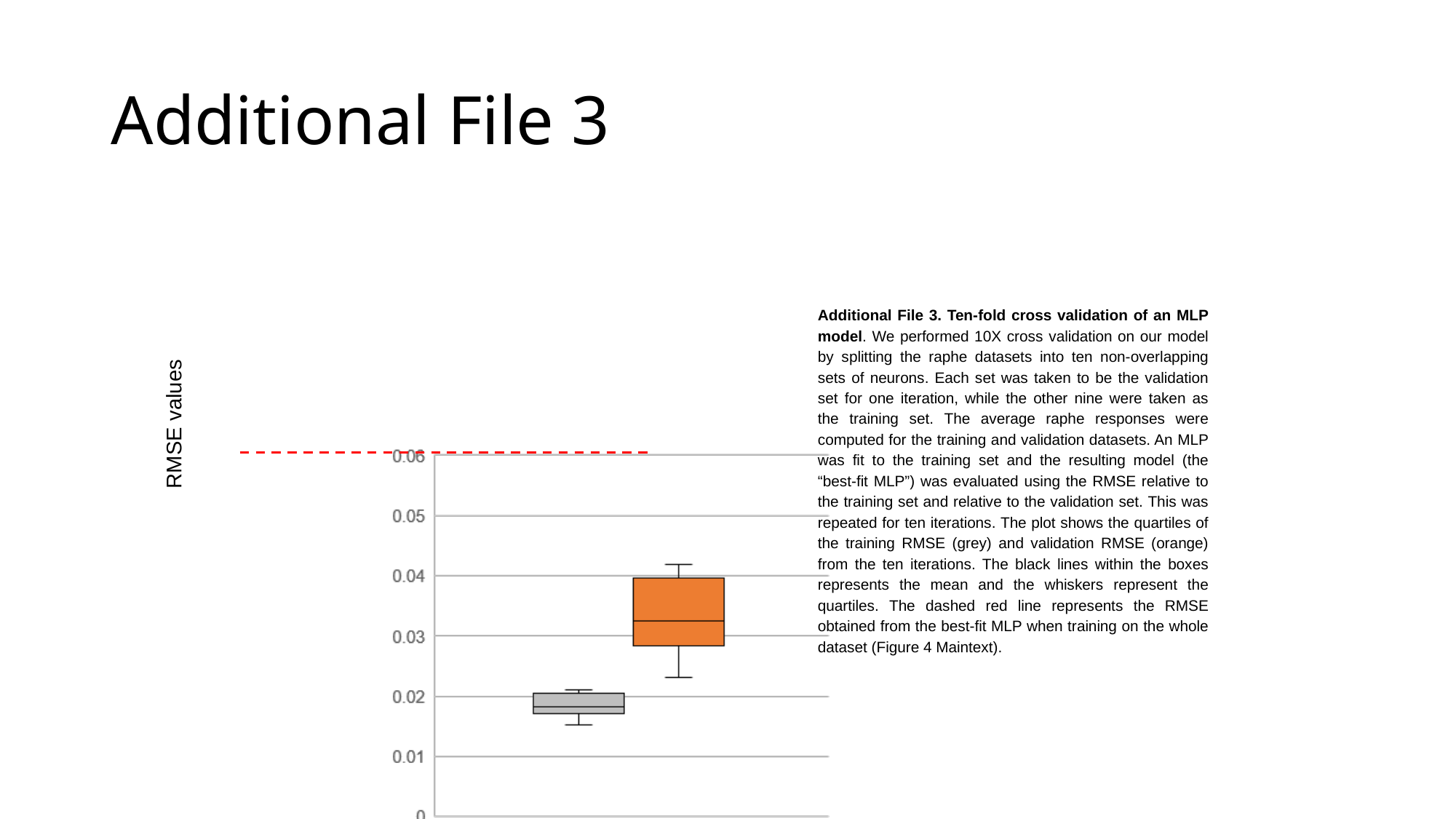

# Additional File 3
Additional File 3. Ten-fold cross validation of an MLP model. We performed 10X cross validation on our model by splitting the raphe datasets into ten non-overlapping sets of neurons. Each set was taken to be the validation set for one iteration, while the other nine were taken as the training set. The average raphe responses were computed for the training and validation datasets. An MLP was fit to the training set and the resulting model (the “best-fit MLP”) was evaluated using the RMSE relative to the training set and relative to the validation set. This was repeated for ten iterations. The plot shows the quartiles of the training RMSE (grey) and validation RMSE (orange) from the ten iterations. The black lines within the boxes represents the mean and the whiskers represent the quartiles. The dashed red line represents the RMSE obtained from the best-fit MLP when training on the whole dataset (Figure 4 Maintext).
RMSE values
